# 2523 Noninvasive biomarkers for inflammatory bowel disease: Drawbacks and potential

**DOI:** 10.1017/cts.2018.102

**Published:** 2018-11-21

**Authors:** Vihang Patel, Sherif Seif, Terrence Barrett

**Affiliations:** 1 University of Kentucky Center for Clinical and Translational Science; 2 University of Kentucky; 3 Department of Internal Medicine, University of Kentucky

## Abstract

OBJECTIVES/SPECIFIC AIMS: Approximately 1.6 million Americans suffer from inflammatory bowel diseases (IBD), ulcerative colitis, and Crohn’s disease. It is a challenge for both physicians and patients alike to manage the disease, primarily due to lack of disease specific biomarkers. Endoscopy remains the gold standard to diagnose and evaluate IBD activity. Current biomarkers or their combinations cannot adequately predict IBD progression or relapse, and response to therapy. METHODS/STUDY POPULATION: In total, 97 IBD patients recruited at University of Kentucky undergoing endoscopy. Patients medical information was collected from electronic database including C-reactive protein (CRP), fecal calprotectin (FC), endoscopy/pathology report. RESULTS/ANTICIPATED RESULTS: The mean CRP and FC levels were 1.3 (normal <1) and 679 (normal <162), respectively. FC (sensitivity 74%) was more reliable to predict mucosal inflammation compare to CRP (sensitivity 36%). However, 52% of patients did not have FC performed (vs. CRP only 4%), and 45% of these patients failed to submit stool sample for analysis. DISCUSSION/SIGNIFICANCE OF IMPACT: Our data suggests FC is the most promising noninvasive marker for disease monitoring in IBD. It correlates well with endoscopic activity and mucosal inflammation. However, further analysis must be done to evaluate barriers to testing and issues with compliance from patients. We feel strongly that a blood biomarker for disease activity is vital for disease monitoring and response to therapy in IBD.

